# COVID-19 disease severity in persons infected with the Omicron variant compared with the Delta variant in Qatar

**DOI:** 10.7189/jogh.12.05032

**Published:** 2022-07-06

**Authors:** Adeel A Butt, Soha R Dargham, Patrick Tang, Hiam Chemaitelly, Mohammad R Hasan, Peter V Coyle, Anvar H Kaleeckal, Ali Nizar Latif, Srusvin Loka, Riyazuddin M Shaik, Ahmed Zaqout, Muna A Almaslamani, Abdullatif Al Khal, Roberto Bertollini, Abdul-Badi Abou-Samra, Laith J Abu-Raddad

**Affiliations:** 1 Hamad Medical Corporation, Doha, Qatar; 2 Department of Medicine, Weill Cornell Medicine, New York, New York; and Doha, Qatar; 3 Department of Population Health Sciences, Weill Cornell Medicine, New York, New York; and Doha, Qatar; 4 Sidra Medicine, Doha, Qatar; 5 Ministry of Public Health Qatar, Doha, Qatar

## Abstract

**Background:**

Understanding the disease severity associated with the Omicron variant of the SARS-CoV-2 virus is important in determining appropriate management strategies at the individual and population levels. We determined the severity of SARS-CoV-2 infection in persons infected with the Omicron vs the Delta variant.

**Methods:**

We identified individuals with SARS-CoV-2 infection with Delta and propensity-score matched controls with Omicron variant infection from the National COVID-19 Database in Qatar. We excluded temporary visitors to Qatar, those with a prior documented infection, those ≤18 years old, and those with <14 days of follow up after the index test positive date. We determined the rates of admission to the hospital, admission to intensive care unit, mechanical ventilation, or death among those infected with the Delta or Omicron variants.

**Results:**

Among 9763 cases infected with the Delta variant and 11 310 cases infected with the Omicron variant, we identified 3926 propensity-score matched pairs. Among 3926 Delta infected, 3259 (83.0%) had mild, 633 (16.1%) had moderate and 34 (0.9%) had severe/critical disease. Among 3926 Omicron infected, 3866 (98.5%) had mild, 59 (1.5%) had moderate, and only 1 had severe/critical disease (overall *P* < 0.001). Factors associated with less moderate or severe/critical disease included infection with Omicron variant (aOR = 0.06; confidence interval (CI) = 0.05-0.09) and vaccination including a booster (aOR = 0.30; 95% CI = 0.09-0.99).

**Conclusions:**

Omicron variant infection is associated with significantly lower severity of disease compared with the Delta variant. Vaccination continues to offer strong protection against severe/critical disease.

The SARS-CoV-2 pandemic has rapidly evolved over time with new viral strains appearing at regular and frequent intervals. The new strains which are associated with significant changes in the behavior of the virus, and/or its effect on the host have been termed “variants of concern” (VOC) and labeled with the Greek alphabets sequentially by the World Health Organization. The Omicron variant is the most recent VOC, which was first identified in South Africa in November 2021, but rapidly spread across the globe and is now the predominant circulating variant in most countries [1-3]. Most Omicron variant infections occur in previously vaccinated persons, and in up to 20% of the cases there is no apparent link to known cases suggesting widespread community transmission [2]. Neutralization studies indicate that the Omicron variant may be more likely to escape immune protection from previous infection compared with the earlier VOCs, which may explain its increased infectiousness [4]. However, early clinical reports from South Africa and North America suggest that the Omicron variant infection may be associated with a lower risk of hospitalization and less severe disease compared with the Delta variant [5-7]. Current vaccines seem effective against severe and/or critical disease due to the Omicron variant, though this effectiveness is lower than the observed effectiveness against earlier variants, and among those who have not received a booster dose [8-10]. There are scant data comparing COVID-19 disease severity in other geographically and ethnically diverse populations infected with the Omicron variant compared with those infected with the Delta variant. We sought to compare the rate of hospitalization, mechanical ventilation, intensive care unit (ICU) admission, and case-fatality among those with Omicron variant infection compared with matched controls with Delta variant infection.

## METHODS

### Study setting

The study was conducted in Qatar, which has one of the highest rates of testing and vaccination of the eligible population for SARS-CoV-2 in the world [11]. Since the identification of the first patient with SARS-CoV-2 on February 27, 2020, Qatar has experienced five distinct waves, now attributed to the wild-type, Alpha, Beta, Delta, and Omicron variants [12-15]. The first case of Omicron variant infection in Qatar was identified on November 24, 2021, and within 4 weeks, it became the predominant strain. Starting very early in the pandemic, Qatar also instituted an aggressive testing policy, which included testing of all persons with compatible symptoms, contacts of confirmed cases, returning travelers, and persons in front-line high-risk professions (eg, health care workers, school staff, salon/spa workers). Multiple general screening campaigns were also carried out, targeting persons in high incidence areas and large convenience samples. RT-qPCR was used to test for SARS-CoV-2 on nasopharyngeal swabs at a single national laboratory at Hamad Medical Corporation, which is accredited by the College of American Pathologists and the Joint Commission International. Surveillance for SARS-CoV-2 variants in Qatar was based on viral genome sequencing and multiplex, real-time reverse-transcription PCR (RT-qPCR) variant genotyping [16] of random positive clinical samples, [14,15,17-19] and complemented by deep sequencing of wastewater samples [19] for the earlier VOCs. Due to the rapid, exponential rise of Omicron variant infections, large scale genome sequencing was not possible in the Omicron pre-dominant era, and positive cases from December 23, 2021 onwards were considered Omicron based on representative sampling described below.

### Study participants

Using the national COVID-19 database in Qatar, which includes all persons tested for SARS-CoV-2 with a RT-PCR test since the beginning of the pandemic, [17,18,20] we identified those with Delta variant infection diagnosed between 23 March and 6 November 2021 and those with Omicron variant infection diagnosed on or after 23 December 2021. We excluded temporary visitors to Qatar, those with a prior documented infection, those under the age of 18, and those with less than 14 days of follow up after the index test positive date. After these exclusions, we propensity-score matched each Delta infection case with an Omicron infection case. The propensity score was estimated based on age, gender, nationality, vaccination status at time of infection, and presence of co-morbidities. We performed 1:1 matching, using the nearest neighbor matching with a caliper of 0.2SD.

### Definitions

The primary outcome of interest was severity of COVID-19 disease in persons infected with the Delta variant compared with those infected with the Omicron variant. Disease severity was categorized into mild (RT-PCR confirmed infection with or without presence of symptoms but not requiring hospitalization), moderate (symptomatic disease requiring acute care hospitalization but no intensive care unit admission or mechanical ventilation), and severe/critical (admission to an intensive care unit, mechanical ventilation, or death). All outcomes within 14 days of the index positive test were included. All patients with COVID-19 in Qatar requiring hospitalization are admitted to designated hospitals within a single health care system (Hamad Medical Corporation), thereby ensuring complete capture of all hospital admissions and subsequent inpatient care. Comorbidities were identified based on associated diagnostic codes in the electronic medical records, as used in our previous publications [21-23]. SARS-CoV-2 infection was confirmed from the national COVID-19 database, which includes every test performed in Qatar since the beginning of the pandemic [17,18,20]. Vaccination status, type of vaccine, and date(s) of administration were also confirmed from the national COVID-19 database, which contains a record of every SARS-CoV-2 vaccine administered in Qatar [17,18].

### Laboratory Methods and Classification by Variant Type

Nasopharyngeal and/or oropharyngeal swabs were collected for PCR testing and placed in Universal Transport Medium (UTM). Aliquots of UTM were extracted on a QIAsymphony platform (QIAGEN, USA) and tested with real-time reverse-transcription PCR (RT-qPCR) using TaqPath COVID-19 Combo Kits (Thermo Fisher Scientific, USA) on an ABI 7500 FAST (Thermo Fisher, USA); tested directly on the Cepheid GeneXpert system using the Xpert Xpress SARS-CoV-2 (Cepheid, USA); or loaded directly into a Roche cobas® 6800 system and assayed with a cobas® SARS-CoV-2 Test (Roche, Switzerland). The first assay targets the viral S, N, and ORF1ab gene regions. The second targets the viral N and E-gene regions, and the third targets the ORF1ab and E-gene regions. All PCR testing was conducted at the Hamad Medical Corporation Central Laboratory following standardized protocols.

Surveillance for SARS-CoV-2 variants in Qatar is based on viral genome sequencing and multiplex, real-time reverse-transcription PCR (RT-qPCR) variant screening^15^ of random positive clinical samples, [13,14,16-18] and complemented by deep sequencing of wastewater samples [18]. The ascertainment of the Delta and Omicron variants in this study was based on the results of the weekly viral genome sequencing and RT-qPCR genotyping of the positive clinical samples [14,18]. The accuracy of the RT-qPCR genotyping was verified against either Sanger sequencing of the receptor-binding domain (RBD) of SARS-CoV-2 surface glycoprotein (S) gene, or by viral whole-genome sequencing on a Nanopore GridION sequencing device. From 108 random positive samples, PCR genotyping was able to assign a genotype in 91 samples: 82 of 82 Delta (B.1.617.2), 9 of 9 Omicron (BA.1/BA.2) were in agreement with sequencing. Of the remaining 17 samples: 9 failed PCR amplification and sequencing, 8 could not be assigned a genotype by PCR (4 of 8 were B.1.617.2 by sequencing, and the remaining 4 failed sequencing). All variant genotyping was conducted at the Sidra Medicine Laboratory following standardized protocols.

### Statistical analyses

Based on an *a priori* assumption that a 25% reduction in rate of hospitalization in those infected with the Omicron variant constitutes a clinically significant difference, we calculated that a sample size of 4008 persons (2004 in the Delta group and 2004 in the Omicron group) would be needed to detect this difference at an alfa level of .05 with a power of 80% if the rate of hospitalization was 10% among those infected with the Delta variant.

We calculated and compared the proportions of persons with mild, moderate, or severe/critical disease among those infected with the Delta and the Omicron variants overall and stratified by vaccination status. 95% confidence intervals (CIs) were calculated to express the spread. Multivariable logistic regression was used to calculate the adjusted odds ratios (aORs) and 95% CIs for factors associated with these outcomes. Where *P* values were used for comparison, a *P* value <0.05 was considered statistically significant. All analyses were done using IBM-SPSS version 27.0 (IBM, Armonk, NY, USA).

### Ethical considerations

The study was approved by the Institutional Review Boards at Hamad Medical Corporation, Weill Cornell Medicine-Qatar, and Qatar University (all in Doha, Qatar). A waiver of informed consent was granted due to the retrospective nature of data retrieval.

## RESULTS

**Figure 1:**
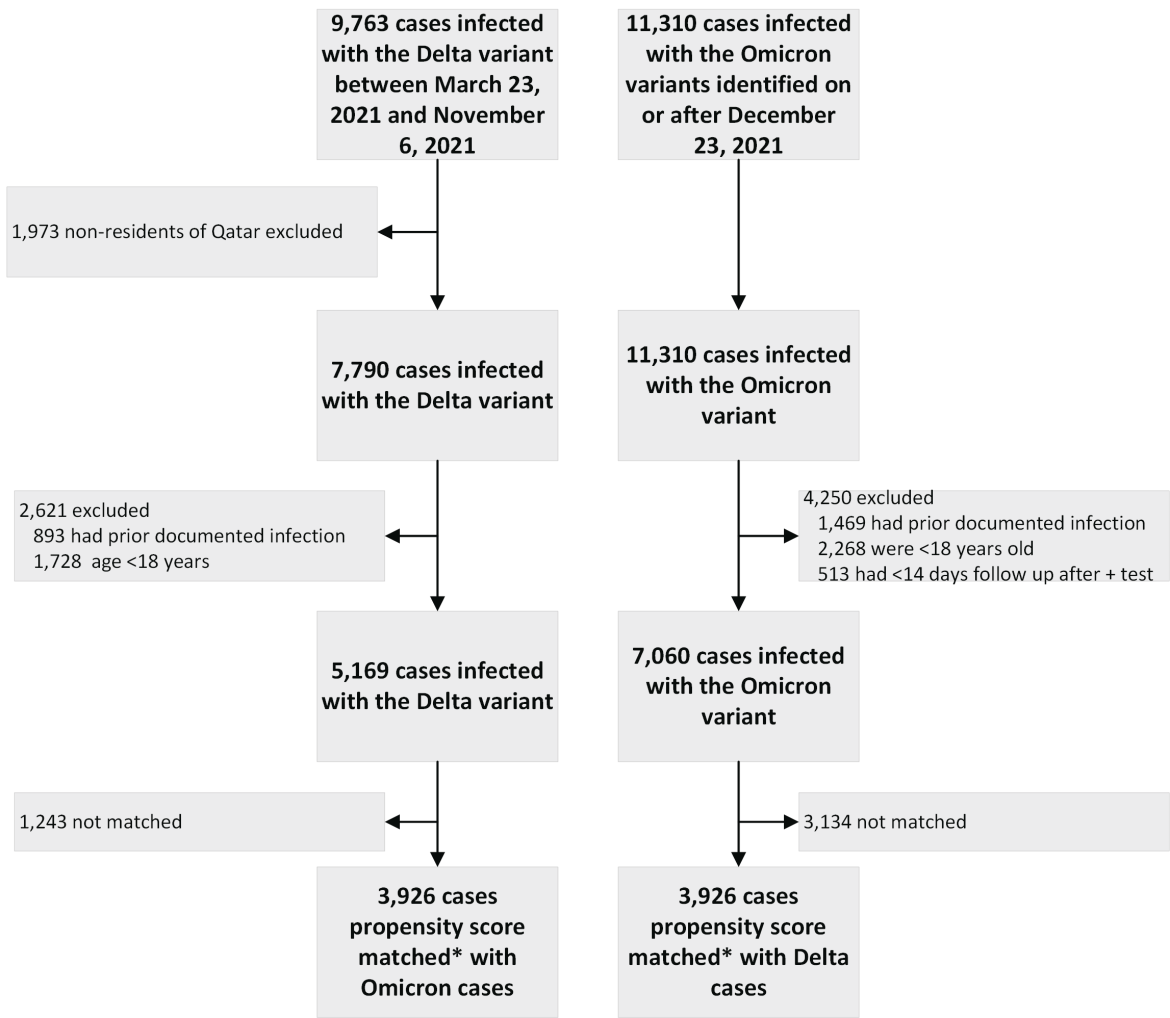
Study flow. *Propensity-score matching done on age, sex, nationality, comorbidities, vaccination status; nearest neighbor matching with caliper of 0.2SD.

Among 9763 cases infected with the Delta variant and 11 310 cases infected with the Omicron variant, we created our analyzable data set consisting of 3926 persons infected with the Delta variant and 3926 propensity-score matched persons infected with the Omicron variant **(****Figure 1****).** For the Delta group, the median age (IQR) was 35.0 (95% CI = 29.0-43.0), 56.2% were male, 29.6% were Qatari nationals, 85.5% had no comorbidities, 66.4% had received at least 2 doses of an mRNA vaccine, and only 0.1% had received a third booster dose >14 days prior to infection. For the Omicron group, the median age (IQR) was 34.0 (26.0-44.0), 53.9% were male, 49.2% were Qatari nationals, 80.4% had no comorbidities, 65.9% had received at least 2 doses of an mRNA vaccine, and 6.4% had also received a third booster dose >14 days prior to infection **(****Table 1****).** Baseline characteristics of the pre-matched study population (5169 with Delta and 7060 with Omicron) are also presented in **Table 1**. Among those with Delta variant infection, 1240 (31.6%) were not vaccinated, 79 (2.0%) had received only 1 dose, 504 (12.8%) had received two doses with the second dose <3 months prior to infection, 2099 (53.5%) had received two doses with the second dose >3 months prior to infection, and 4 (0.1%) had also received a third dose >14 days prior to infection. Among those with Omicron variant infection, 1317 (33.5%) were not vaccinated, 23 (0.6%) had received only 1 dose, 36 (0.9%) had received two doses with the second dose <3 months prior to infection, 2300 (58.6%) had received two doses with the second dose >3 months prior to infection, and 250 (6.4%) had also received a third dose >14 days prior to infection (Table S1 in the **Online Supplementary Document**). A comparison of baseline characteristics of the full cohort of persons infected with the Delta and Omicron variants before exclusions for prior infection or age <18 years is presented in Table S2 in the **Online Supplementary Document**.

**Table 1:** Baseline characteristics of propensity score matched persons infected with the Delta and Omicron variants

	Before propensity score matching			After propensity score matching		
	**Delta variant, N = 5169 (N, %)**	**Omicron variant, N = 7060 (N, 5)**	SMD*	**Delta variant, N = 3926 (N, %)**	**Omicron variant, N = 3926 (N, 5)**	SMD*
**Age, median (IQR)**	35.0 (29.0-42.0)	34.0 (28.0-42.0)	0.041	35.0 (29.0-43.0)	34.0 (26.0-44.0)	0.044
**Age (years)**						
≤35	1458 (28.2)	2120 (30)	0.039	2034 (51.8)	2122 (54.0)	0.132
36-45	2030 (39.3)	2756 (39)		1176 (30.0)	968 (24.7)	
46-55	1130 (21.9)	1468 (20.8)		496 (12.6)	552 (14.1)	
56-65	414 (8.0)	551 (7.8)		190 (4.8)	237 (6.0)	
66+	137 (2.7)	165 (2.3)		30 (0.8)	47 (1.2)	
**Sex**						
Female	2139 (41.4)	3047 (43.2)	0.036	1720 (43.8)	1811 (46.1)	0.047
Male	3030 (58.6)	4013 (56.8)		2206 (56.2)	2115 (53.9)	
**Nationality**						
Qatari	1340 (25.9)	2039 (28.9)	0.074	1164 (29.6)	1931 (49.2)	0.413
Craft labor nationalities	2103 (40.7)	2659 (37.7)		1386 (35.3)	1079 (27.5)	
Other‡	1726 (33.4)	2362 (33.5)		1376 (35.0)	916 (23.3)	
**Comorbidities**						
Hypertension	385 (7.4)	516 (7.3)	0.005	302 (7.7)	358 (9.1)	0.051
Chronic lung disease	218 (4.2)	364 (5.2)	0.044	170 (4.3)	296 (7.5)	0.136
Cardiovascular disease	324 (6.3)	427 (6.0)	0.009	259 (6.6)	319 (8.1)	0.059
Diabetes	254 (4.9)	379 (5.4)	0.021	205 (5.2)	293 (7.5)	0.092
Cancer	24 (0.5)	36 (0.5)	0.007	17 (0.4)	26 (0.7)	0.031
Chronic kidney disease	6 (0.1)	15 (0.2)	0.023	5 (0.1)	8 (0.2)	0.019
Chronic liver disease	6 (0.1)	10 (0.1)	0.007	4 (0.1)	5 (0.1)	0.008
Stroke	11 (0.2)	11 (0.2)	0.013	8 (0.2)	8 (0.2)	0.000
Autoimmune disease	14 (0.3)	22 (0.3)	0.008	11 (0.3)	15 (0.4)	0.018
**Comorbidities count**						
None	4447 (86.0)	6005 (85.1)	0.028	3358 (85.5)	3155 (80.4)	0.139
1 comorbidity	389 (7.5)	577 (8.2)		300 (7.6)	420 (10.7)	
2+ comorbidities	333 (6.4)	478 (6.8)		268 (6.8)	351 (8.9)	
**Vaccination status at time of infection**						
Not Vaccinated or only 1 dose	2562 (49.6)	1342 (19.0)	0.680	1319 (33.6)	1340 (34.1)	0.011
At least 2 doses	2607 (50.4)	5718 (81.0)		2607 (66.4)	2586 (65.9)	

IQR – interquartile range; SMD – standardized mean difference

*Standardized mean difference of <0.1 suggest adequate matching.

†Includes India, Pakistan, Bangladesh, Nepal, Sri Lanka, and Sudan due to large proportions of these nationals being craft and manual workers.

‡Other nationalities include 44 nationalities.

Among those infected with the Delta variant, 3259 (83.0%) had mild/asymptomatic disease while 633 (16.1%) fulfilled the criteria for moderate infection. Among those infected with the Omicron variant, 3866 (98.5%) had mild/asymptomatic disease, while 59 (1.5%) fulfilled the criteria of moderate infection within 14 days of the positive index SARS-CoV-2 test. There were 34 cases of severe/critical disease among those infected with the Delta variant and only 1 case among those infected with the Omicron variant (overall *P* < 0.001; **Table 2**). Of the moderate or severe/critical infections in the Delta variant group, none (0%) occurred in those who had received a booster dose at 14 days prior. Of the 59 moderate or severe/critical infections in the Omicron variant group, only 3 (1.2%) were among those who had received a booster dose of the mRNA vaccine at least 14 days prior to the infection. All three had moderate infection **(****Table 3****).**

**Table 2:** Summary of disease outcomes (%, 95 CI) of the two SARS-CoV-2 variant groups

	Delta variant, N = 3926	Omicron variant, N = 3926	*P*-value
Outcome-disease status*			
Mild	3259 (83.0)	3866 (98.5)	<0.001
Moderate	633 (16.1)	59 (1.5)	
Severe/critical	34 (0.9)	1 (0.0)	
Moderate or severe/critical outcome	667 (17.0)	60 (1.5)	<0.001

CI – confidence interval

*Mild: infection confirmed but no hospitalization; Moderate: hospitalized but no ICU admission or mechanical ventilation; Severe/critical: Mechanical ventilation or ICU admission or death.

**Table 3:** Summary of disease outcomes (%, 95% CI) of the two SARS-CoV-2 variant groups, stratified by vaccination status.

	Infection among those with no booster (post 14 d of 2^nd^ dose up to14 d post 3^rd^ dose)			Infection among those with booster (Infection after 14 d of 3^rd^ dose)		
	**Delta, N = 3922**	**Omicron, N = 3676**	***P*-value**†	**Delta, N = 4**	**Omicron, N = 253**	***P*-value**‡
Outcome-disease status*						
Mild	3255 (83.0)	3619 (98.4)	<0.001	4 (100.0)	247 (98.8)	>0.999
Moderate	633 (16.1)	56 (1.5)		0 (0.0)	3 (1.2)	
Severe-critical	34 (0.9)	1 (0.0)		0 (0.0)	0 (0.0)	
Moderate or severe/critical	667 (17.0)	57 (1.5)	<0.001	0 (0.0)	3 (1.2)	>0.999

CI – confidence interval

*Mild: infection confirmed but no hospitalization; Moderate: hospitalized but no ICU admission or mechanical ventilation; Severe/critical: Mechanical ventilation OR ICU admission OR death.

†*P*-value estimated using the χ^2^ test.

‡*P*-value estimated using the Fisher Exact test.

In the multivariable logistic regression model, the adjusted odds ratio (aOR) for the Omicron variant infection to develop an adverse outcome (either moderate or severe/critical disease) was 0.06 (95% CI = 0.05-0.09) The odds of an adverse outcome were higher with increasing age and increasing number of comorbidities. Male sex was associated with lower odds of an adverse outcome (aOR = 0.73, 95% CI = 0.61-0.87). **(****Table 4****)** The breakdown by moderate and severe/critical disease is also provided in **Table 4**. The factors associated with developing moderate or severe/critical disease stratified by the variant causing infection were generally similar when results were stratified by the variant causing infection, through some of the associations were not statistically significant due to smaller numbers. (Table S3 in the **Online Supplementary Document**) A numerical breakdown of disease severity by vaccination status, stratified by the variant causing infection is also provided in Table S4 in the **Online Supplementary Document**.

**Table 4:** Multivariable logistic regression with outcome disease status as dependent variable

	Either moderate or severe/critical disease*		Moderate disease*		Severe/critical disease*	
	**aOR (95% CI)**	***P*-value**	**aOR (95% CI)**	***P*-value**	**aOR (95% CI)**	***P*-value**
**Omicron variant** (comparator: Delta variant)	0.065 (0.049-0.086)	<0.001	0.067 (0.051-0.090)	<0.001	0.019 (0.003-0.143)	<0.001
**Vaccination status at time of infection** (comparator: Not vaccinated at time of infection)						
Vaccinated with only 1 dose at time of infection	1.26 (0..70-2.29)	0.442	1.26 (0.69-2.234)	0.454	1.11 (0.14-8.93)	0.923
Vaccinated with 2^nd^ dose <3 months prior to infection	0.66 (0.48-0.90)	0.009	0.69 (00.50-0.95)	0.023	0.18 (0.04-0.86)	0.031
Vaccinated with 2^nd^ dose >3 months prior to infection	0.86 (0.70-1.06)	0.164	0.91 (0.74-1.13)	0.390	0.24 (0.10-0.56)	0.001
Vaccinated with 3^rd^ dose >14 days prior to infection	0.30 (0.09-0.99)	0.049	0.33 (0.10-1.08)	0.067	N/A	N/A
**Age** (comparator: ≤35 years)						
36-45	1.35 (1.09-1.67)	0.0.005	1.31 (1.05-1.62)	0.015	2.88 (1.09-7.65)	0.033
46-55	4.70 (3.73-5.91)	<0.001	4.63 (3.67-5.85)	<0.001	7.32 (2.51-31.39)	<0.001
56-65	3.10 (2.17-4.41)	<0.001	2.90 (2.02-4.17)	<0.001	10.08 (2.74-37.11)	<0.001
+66	7.65 (3.93-14.89)	<0.001	7.16 (3.62-14.16)	<0.001	48.65 (6.72-352.17)	<0.001
**Male sex** (comparator: female)	0.73 (0.61-0.87)	<0.001	0.71 (0.59-0.85)	<0.001	1.55 (0.71-3.41)	0.272
**Nationality** (comparator: Qatari)						
Craft and manual worker nationalities	0.95 (0.75-1.20)	0.673	0.94 (0.74-1.19)	0.598	1.39 (0.50-3.83)	0.527
Other nationalities	1.37 (1.11-1.69)	0.004	1.37 (1.11-1.70)	0.004	1.56 (0.58-4.23)	0.382
**Comorbidities count** (comparator: zero)						
1-2	1.56 (1.18-2.07)	0.002	1.48 (1.10-1.97)	0.009	4.90 (1.75-13.74)	0.003
3 or more	2.06 (1.55-2.74)	<0.001	2.01 (1.50-2.69)	<0.001	4.37 (1.46-13.08)	0.008

CI – confidence interval, aOR – adjusted odds rato

*Moderate: hospitalized but no ICU admission or mechanical ventilation; Severe/critical: Mechanical ventilation or ICU admission or death.

## DISCUSSION

Early reports suggest that persons infected with the SARS-CoV-2 Omicron variant may be at a lower risk for adverse outcomes compared with the earlier VOCs. We provide results from a national study describing clinical outcomes in persons infected with the Omicron variant compared with propensity score matched controls infected with the Delta variant.

We found that persons infected with the Omicron variant were significantly less likely to experience moderate or severe/critical disease in the 14-day period after diagnosis compared with those infected with the Delta variant. Persons infected with the Omicron variant were nearly 11-fold less likely to require hospital admission compared with those infected with the Delta variant. There was almost no severe/critical disease in persons with Omicron variant infection (only 1 person in this group). We recently reported the severity of infection among patients infected with the Delta variant compared with those infected with the Beta variant. In that study, those with Delta infection were more likely to be hospitalized compared with propensity-score matched persons infected with the Beta variant (27.3% vs 20.0%, *P* = 0.01).[24] The reason for lesser severity with Omicron variant infection is not yet known. It is possible that the large number of novel mutations may have attenuated the virulence of this variant. In vitro studies indeed suggest that Omicron variant has lower efficiency of replication and fusion compared with the Delta variant, which may partly explain these findings [25]. In addition, the high background vaccination rates among the population may have ameliorated its severity.

The vast majority of moderate or severe/critical disease events occurred in persons who had not received a booster dose of the vaccine. While this was true for both Omicron and Delta variant infections, the difference was more pronounced in the Delta variant infections. Among those who had received a booster dose, there were only 3 cases of moderate disease (all among Omicron group), and no cases of severe/critical disease with either variant. This suggests that a booster dose is highly protective against moderate and severe/critical disease, though the effect is somewhat attenuated in those with Omicron variant infection.

Increasing age and presence of comorbidities were strongly associated with moderate and severe-critical disease even after adjusting for the variant causing the infection and the vaccination status. These are well-known risk factors for poor outcomes in patients with SARS-CoV-2 infection. These factors were associated with a higher risk of poor outcomes in our study population.

It is important to note that while infection with the Omicron variant may be associated with less severe disease, it remains a potent threat due to its enhanced transmission potential and the large number of resultant infections. Due to this large burden of infections, even lower rates of hospitalizations and the need for intensive care may overwhelm the already stretched health care capacity [26]. In the US, the Omicron variant infections have already caused higher increases in daily death counts compared with the previous variants [27].

Strengths of our study include a large national population, extensive testing, a single testing site, and uniform data collection methods. Since all patients requiring inpatient care are admitted to a single health care system by the same group of physicians, there is a high degree of uniformity in admission criteria and subsequent care, including decisions to transfer to an intensive care setting, and initiation of mechanical ventilation. To mitigate potential bias from an imbalance in baseline risk factors, we conducted a robust propensity score matching of the two study groups. Certain limitations also need to be noted. This was a retrospective study. Variant identification was based on RT-qPCR variant genotyping and only a subset were subjected to whole genome sequencing. However, we have demonstrated a high level of correlation between genotyping and viral sequencing. Since the two population groups were from different time periods, it is possible to introduce a bias due to changing exposure and vaccination status at different time points in the pandemic. However, we addressed this by excluding those with a documented history of infection and stratifying the results by vaccination status. Since we used the Qatar National COVID-19 database, which has a record of every PCR test and every vaccine administered in Qatar, we are very confident that we captured all prior documented infections and vaccination.

## CONCLUSIONS

In conclusion, infection with the Omicron variant is associated with significantly lower severity of infection as measured by hospitalization rates and need for intensive care unit care or mechanical ventilation. Vaccination continues to offer strong protection against severe/critical disease.

## Additional material


Online Supplementary Document
