# Supplementary material for: COVID-19 disease severity in persons infected with the Omicron variant compared with the Delta variant in Qatar
Source: J Glob Health. 2022 Jul 6;12:05032. doi: 10.7189/jogh.12.05032 (PMC9253930; doi:10.7189/jogh.12.05032)
Supplement: Online Supplementary Document [file jogh-12-05032-s001.pdf]

Supplementary Data

Title: **COVID-19 Disease Severity in Persons Infected with the Omicron Variant**

Adeel A. Butt, MBBS, MS;[1,2,3] Soha R. Dargham, MPH;[3] Patrick Tang, MD, PhD;[4] Hiam Chemaitelly, MS;[3] Mohammad R. Hasan, PhD;[2,3] Peter V. Coyle, MD;[1] Anvar H. Kaleeckal, MS;[1] Ali Nizar Latif, MD;[1] Srusvin Loka,BCA;[1] Riyazuddin M. Shaik, MA, MHM;[1] Ahmed Zaqout, MD;[1] Muna A. Almaslamani, MD;[1] Abdullatif Al Khal, MD;[1] Roberto Bertollini, MD, MPH;[5] Abdul-Badi Abou-Samra, MD, PhD;[1] Laith J. Abu-Raddad, PhD;[3]*

Table of Contents:

Supplementary table 1. Details regarding the time of vaccination in relation to the time of infection.

Supplementary table 2. Baseline characteristics of the full cohort of persons infected with the Delta and Omicron variants before exclusions for prior infection or age <18 years.

Supplementary table 3. Multivariable logistic regression with outcome disease status as dependent variable, stratified by variant.

Supplementary table 4. Disease severity distribution by vaccination status, stratified by variant.

Supplementary table 1. Details regarding the time of vaccination in relation to the time of infection.

	Before propensity score matching			After propensity score matching		
	Delta N=5,169	Omicron N=7,060	SMD	Delta N=3,926	Omicron N=3,926	SMD
	N (%)	N (%)		N (%)	N (%)	
**Vaccination status at time of infection**						
Not vaccinated	2405 (46.5)	1319 (18.7)	0.930	1240 (31.6)	1317 (33.5)	0.616
Only 1 dose	157 (3.0)	23 (0.3)		79 (2.0)	23 (0.6)	
2nd dose <3 months prior to infection	504 (9.8)	131 (1.9)		504 (12.8)	36 (0.9)	
2nd dose > 3 months prior to infection	2099 (40.6)	5021 (71.1)		2099 (53.5)	2300 (58.6)	
3rd dose >14 days prior to infection	4 (0.1)	566 (8.0)		4 (0.1)	250 (6.4)	

Supplementary table 2. Baseline characteristics of the full cohort of persons infected with the Delta and Omicron variants before exclusions for prior infection or age <18 years.

	**Omicron variant N=11,310**	**Delta variant N=7,790**	**Standard difference**
	**% (95% CI)**	**% (95% CI)**	
**Age**			
Median (IQR), years	34.0 (28.0-41.0)	35.0 (29.0-42.0)	0.065
18-29 years	5130 (45.4)	3555 (45.6)	0.016
30-39 years	3470 (30.7)	2352 (30.2)	
40-49 years	1823 (16.1)	1280 (16.4)	
50-59 years	678 (6)	453 (5.8)	
+60 years	209 (1.8)	150 (1.9)	
**Sex**			
Female	4923 (43.5)	3217 (41.3)	0.045
Male	6387 (56.5)	4573 (58.7)	
**Nationality**			
Qatari	4040 (35.7)	2500 (32.1)	0.081
Craft and manual worker nationalities*	3706 (32.8)	2778 (35.7)	
Other nationalities&	3564 (31.5)	2512 (32.2)	
**Comorbidities**			
Hypertension	643 (5.7)	433 (5.6)	0.005
Diabetes	470 (4.2)	288 (3.7)	0.024
Chronic lung disease	898 (7.9)	550 (7.1)	0.033
Cardiovascular disease	558 (4.9)	359 (4.6)	0.015
Chronic kidney disease	20 (0.2)	7 (0.1)	0.024
Chronic liver disease	14 (0.1)	9 (0.1)	0.002
Cancer diagnosis	45 (0.4)	27 (0.3)	0.008
Autoimmune disease	32 (0.3)	15 (0.2)	0.019
Stroke	13 (0.1)	11 (0.1)	0.007
**Comorbidity count**			
None	9528 (84.2)	6671 (85.6)	0.039
1	1180 (10.4)	746 (9.6)	
2 or more	602 (5.3)	373 (4.8)	
**Vaccination status at time of infection**			
Not vaccinated at time of infection	3642 (32.2)	4612 (59.2)	0.814
Vaccinated with only 1 dose at time of infection	51 (0.5)	207 (2.7)	
Vaccinated with 2nd dose 3 months prior to infection	246 (2.2)	632 (8.1)	
Vaccinated with 2nd dose > 3 months prior to infection	6677 (59)	2335 (30)	
Vaccinated with 3rd dose >14 days prior to infection	694 (6.1)	4 (0.1)	
**Infection status in relation to vaccination**			
Infection before or up to 14 days of 2nd dose	3709 (32.8)	4844 (62.2)	0.677
Infection post 14 days of 2nd dose up to 14 days post 3rd dose	6907 (61.1)	2942 (37.8)	
Infection >14 days after 3rd dose	694 (6.1)	4 (0.1)	

*Includes India, Pakistan, Bangladesh, Nepal, Sri Lanka, and Sudan due to large proportions of these nationals being craft and manual workers.

†Other nationalities include 44 nationalities.

Supplementary table 3. Multivariable logistic regression with outcome disease status as dependent variable, stratified by variant.

	**Moderate or severe/critical disease**			**Moderate disease***			**Severe/Critical disease***		
	**Omicron**	**Delta**		**Omicron**	**Delta**		**Omicron†**	**Delta**	
	**aOR (95% CI)**	**aOR (95% CI)**	**P-value$**	**aOR (95% CI)**	**aOR (95% CI)**	**P-value$**	**aOR (95% CI)**	**aOR (95% CI)**	**P-value$**
**Vaccination status at time of infection** (comparator: Not vaccinated at time of infection)									
only 1 dose	N/A	1.14 (0.74-1.75)	N/A	N/A	1.22 (0.79-1.88)	N/A	N/A†	0.40 (0.05-2.97)	N/A†
2nd dose <3 months prior	N/A	0.57 (0.43-0.76)	N/A	N/A	0.63 (0.47-0.84)	N/A	N/A†	0.11 (0.03-0.47)	N/A†
2nd dose > 3 months prior	0.57 (0.31-1.08)	0.67 (0.57-0.80)	0.287	0.62 (0.32-1.18)	0.74 (0.62-0.88)	0.342	N/A†	0.13 (0.07-0.25)	N/A†
3rd dose >14 days prior	0.35 (0.12-0.98)	N/A	N/A	0.33 (0.11-0.99)	N/A	N/A	N/A†	N/A	N/A†
**Age** (comparator: 20-29 years)									
30-39 years	1.38 (0.78-2.46)	1.10 (0.89-1.36)	0.890	1.34 (0.75-2.39)	1.06 (0.85-1.31)	0.867	N/A†	2.88 (0.96-8.67)	N/A†
40-49 years	1.40 (0.71-2.76)	5.57 (2.07-3.19)	0.056	1.40 (0.71-2.74)	2.41 (1.93-3.00)	0.087	N/A†	9.41 (3.22-27.47)	N/A†
50-59 years	1.10 (0.45-2.68)	4.77 (3.63-6.27)	<.006	1.11 (0.46-2.70)	4.44 (3.36-5.87)	0.012	N/A†	18.56 (5.73-60.14)	N/A†
60+ years	1.83 (0.67-4.99)	6.71 (4.44-10.14)	0.163	1.36 (0.46-4.01)	5.85 (3.82-8.97)	0.130	N/A†	42.07 (11.21-157.90)	N/A†
**Male sex** (comparator: female)	0.70 (0.44-1.12)	0.62 (0.52-0.72)	0.621	0.65 (0.41-1.05)	0.59 (0.50-0.69)	0.781	N/A†	1.28 (0.73-2.23)	N/A†
**Nationality** (comparator: Qatari)									
Craft and manual worker nationalities	0.46 (0.25-0.84)	0.78 (0.63-0.97)	0.309	0.43 (0.23-0.80)	0.79 (0.63-0.99)	0.195	N/A†	0.71 (0.33-1.55)	N/A†
Other nationalities	0.47 (0.37-0.82)	1.23 (1.01-1.50)	0.011	0.46 (0.27-0.81)	1.21 (0.99-1.48)	0.013	N/A†	1.70 (0.84-3.43)	N/A†
**Comorbidities count** (comparator: zero)									
1	2.25 (1.20-4.21)	1.83 (1.43-2.35)	0.862	2.25 (1.20-4.23)	1.73 (1.33-2.23)	0.667	N/A†	3.89 (1.83-8.26)	N/A†
2 or more	4.09 (2.10-7.96)	2.41 (1.85-3.15)	0.778	3.94 (2.00-7.76)	2.23 (1.69-2.95)	0.859	N/A†	7.23 (3.46-15.10)	N/A†

*Mild/asymptomatic: infection confirmed but no hospitalization; Moderate: hospitalized but no ICU admission or mechanical ventilation; Severe/critical: Mechanical ventilation OR ICU admission OR death

$p-value comparing odds ratios between Delta and Omicron variants

†Not applicable as there is only 1 severe/critical case among Omicron infections not vaccinated at time of infection; 1 severe/critical case among Omicron infections vaccinated with 2nd dose > 3 months prior to infection; and 1 severe/critical case among Omicron infections vaccinated with 3rd dose >14 days prior to infection

Supplementary table 4. Disease severity distribution by vaccination status, stratified by variant.

		**Mild/no hospitalization**	**Moderate disease**	**Severe/critical disease**
	**Vaccination status at time of infection**	**N**	**N**	**N**
**Delta**	Not vaccinated	1055	170	15
	only 1 dose at time of infection	64	14	1
	2nd dose <3 months prior to infection	435	67	2
	2nd dose > 3 months prior to infection	1701	382	16
	3rd dose >14 days prior to infection	4	0	0
**Omicron**	Not vaccinated	1303	13	1
	only 1 dose at time of infection	23	0	0
	2nd dose <3 months prior to infection	36	0	0
	2nd dose > 3 months prior to infection	2257	43	0
	3rd dose >14 days prior to infection	247	3	0
